# Comparison of the effects of lidocaine and amiodarone for out-of-hospital cardiac arrest patients with shockable rhythms: a retrospective observational study from a multicenter registry

**DOI:** 10.1186/s12872-022-02920-2

**Published:** 2022-11-05

**Authors:** Yuki Kishihara, Masahiro Kashiura, Shunsuke Amagasa, Fumihito Fukushima, Hideto Yasuda, Takashi Moriya

**Affiliations:** 1 Department of Emergency and Critical Care Medicine, Saitama Medical Center, Jichi Medical University, 1-847 Amanuma-cho, Omiya-ku, Saitama-shi, 330-8503 Saitama, Japan; 2 Division of Emergency and Transport Services, National Center for Child Health and Development, Tokyo, Japan; 3 Department of Clinical Research Education and Training Unit, Keio University Hospital Clinical and Translational Research Center, Tokyo, Japan

**Keywords:** Amiodarone, Lidocaine, Out-of-hospital cardiac arrest, Shockable rhythm

## Abstract

**Background:**

Out-of-hospital cardiac arrest (OHCA) with shockable rhythms, including ventricular fibrillation and pulseless ventricular tachycardia, is associated with better prognosis and neurological outcome than OHCA due to other rhythms. Antiarrhythmic drugs, including lidocaine and amiodarone, are often used for defibrillation. This study aimed to compare the effects of lidocaine and amiodarone on the prognosis and neurological outcome of patients with OHCA due to shockable rhythms in a real-world setting.

**Methods:**

We conducted a retrospective observational study using a multicenter OHCA registry of 91 participating hospitals in Japan. We included adult patients with shockable rhythms, such as ventricular fibrillation and pulseless ventricular tachycardia, who were administered either lidocaine or amiodarone. The primary outcome was 30-day survival, and the secondary outcome was a good neurological outcome at 30 days. We compared the effects of lidocaine and amiodarone for patients with OHCA due to shockable rhythms for these outcomes using logistic regression analysis after propensity score matching (PSM).

**Results:**

Of the 51,199 patients registered in the OHCA registry, 1970 patients were analyzed. In total, 105 patients (5.3%) were administered lidocaine, and 1865 (94.7%) were administered amiodarone. After performing PSM with amiodarone used as the reference, the odds ratios and 95% confidence intervals of lidocaine use for 30-day survival and 30-day good neurological outcome were 1.44 (0.58–3.61) and 1.77 (0.59–5.29), respectively.

**Conclusion:**

The use of lidocaine and amiodarone for patients with OHCA due to shockable rhythms within a real-world setting showed no significant differences in short-term mortality or neurological outcome. There is no evidence that either amiodarone or lidocaine is superior in treatment; thus, either or both drugs could be administered.

**Supplementary Information:**

The online version contains supplementary material available at 10.1186/s12872-022-02920-2.

## Background

Despite advancements in medical technology, out-of-hospital cardiac arrest (OHCA) is responsible for numerous deaths, including approximately 300,000–400,000 deaths per year in Europe and the United States [1–3]. Shockable rhythms, including ventricular fibrillation (VF) and pulseless ventricular tachycardia (VT), account for approximately 20–30% of OHCA occurrences [4]. OHCA with shockable rhythms is associated with a better prognosis than OHCA due to other rhythms; for example, OHCA with shockable rhythms is associated with an approximately 30% 30-day survival rate [5, 6]. Although the data are based on patients with in-hospital cardiac arrest, a previous study found that patients with shockable rhythms may also experience better neurological recovery (i.e., a neurological recovery of approximately 50%) than those with non-shockable rhythms [7]. However, long durations of recurrent VF or pulseless VT may lead to poor mortality and neurological outcomes [8, 9]. Therefore, early defibrillation is important for the effective treatment of OHCA with shockable rhythms.

Treatments for VF and pulseless VT include electrical defibrillation and antiarrhythmic drugs. Antiarrhythmic drugs are often used because electrical defibrillation alone often does not restore sustained circulation [10]. These drugs are differentially prescribed according to the patient’s pathophysiology [11]. Among these drugs, lidocaine and amiodarone are commonly used and are strongly recommended in the guidelines [11–13]. Amiodarone has multiple effects, including β-blockage and blockade of depolarizing sodium currents and potassium channels; these actions may be more effective at inhibiting or terminating arrhythmia by influencing automaticity and re-entry compared to other antiarrhythmic drugs with a single mechanism [11]. The latest American Heart Association (AHA) and European Resuscitation Council (ERC) guidelines revised recommendations about amiodarone and lidocaine use for VF and pulseless VT according to the results of a previous randomized control trial (RCT) published in 2016 [12–14]. This RCT compared the use of amiodarone and lidocaine in patients with non-traumatic cardiac arrest with shockable rhythms and reported no significant differences in mortality and neurological outcomes between the two groups [14]. Based on recent studies and revised guidelines, it is likely that the real-world use of antiarrhythmic drugs, including lidocaine and amiodarone, may have changed in recent years [12–14]. However, to the best of our knowledge, there are no studies comparing lidocaine and amiodarone for patients with OHCA due to shockable rhythms in real-world data.

Therefore, it is necessary to compare lidocaine and amiodarone by evaluating clinical outcomes in patients with OHCA due to VF and pulseless VT within a real-world setting. The results of a comparative evaluation of the effects of lidocaine and amiodarone on mortality and neurological outcomes in a real-world setting may have significant implications for verifying external validity in populations other than the ideal populations evaluated within the RCT [14]. Therefore, this study aimed to compare the effects of lidocaine and amiodarone on survival and neurological outcomes in a real-world setting.

## Methods

### Study design

We conducted a multicenter, retrospective observational study using the OHCA registry administered by the Japanese Association for Acute Medicine (JAAM). More specifically, this is a registry of patients with OHCA who were transported to 91 hospitals in Japan between June 1, 2014, and December 31, 2019. This registry collects pre-hospital and post-hospital information on patients with OHCA in Japan. Pre-hospital information was collected from the All-Japan Utstein Registry of the Fire and Disaster Management Agency; the details of this registry were reported in 2010 [15]. Post-hospitalization information was collected by medical personnel, including physicians, at each institution. Pre-hospital and post-hospital information is registered in a web-based system. Information on extraction factors is not stripped or concealed as the physicians supervising study implementation collect these data at each center, and the outcome assessors are not blinded.

Approval for the collection of JAAM-OHCA information was obtained from the ethics committee at each participating hospital. Approval for conducting this study (i.e., secondary analysis) was obtained from the ethics committee of the Jichi Medical University Saitama Medical Centre (approval number: S19–016).

Since no interventions that deviated from general cardiopulmonary resuscitation (CPR) practices were performed for the patients with OHCA evaluated in the current registry-based study, the typical requirement for informed consent was waived by the ethics review committee of each participating institution. However, together with other institutions, we provided an opt-out procedure on the website of the department of emergency medicine of Jichi Medical University Saitama Medical Centre.

This study was conducted according to the guidelines specified within the STrengthening the Reporting of OBservational studies in Epidemiology (STROBE) statement as well as the principles of the Declaration of Helsinki and its later amendments (e-Table 1 in Additional file 1) [16].

### Participants

This study included all patients who met the following criteria: 1) patients with OHCA and 2) patients for whom resuscitation was performed by emergency medical services (EMS) personnel. We included patients who received amiodarone or lidocaine during resuscitation; we attributed lidocaine or amiodarone use to the presence of VF or pulseless VT. Exclusion criteria were as follows: 1) cause of OHCA was not cardiogenic, 2) < 18 years of age, 3) both lidocaine and amiodarone were administered during resuscitation, 4) neither lidocaine nor amiodarone was administered during resuscitation, 5) uncertainty about patient’s use of lidocaine and amiodarone, and 6) missing outcomes for the primary and secondary endpoints described below. We did not exclude based on initial rhythm at cardiac arrest.

### Data collection

The following data were collected: age, sex, transportation by vehicular or air ambulance with a physician present, witness status (none, EMS personnel, others), bystander CPR, the initial monitored cardiac rhythm (VF, pulseless VT, pulseless electrical activity [PEA], asystole, other), the cause of cardiac arrest (cardiogenic, presumed cardiogenic), the presence of shock delivery, administration of adrenaline, antiarrhythmic drug use (amiodarone, lidocaine, nifekalant, magnesium), the time from the emergency call to EMS contact, the time from the emergency call to physician contact, the time from the emergency call to the return of spontaneous circulation (ROSC), the use of extracorporeal membrane oxygenation (ECMO), intra-aortic balloon pumping (IABP), percutaneous coronary intervention (PCI), targeted temperature management (TTM), 30-day survival, and cerebral performance category (CPC) [17].

The data collection was unmasked as the physicians in charge of this investigation collected the data individually, and the outcome assessors were unblinded.

### Outcome measures

The primary outcome evaluated in this study was 30-day survival. The secondary outcome was a good neurological outcome 30 days after cardiac arrest. A good neurological outcome was defined as a CPC score of 1 or 2 [17]. These outcomes were decided upon referring to a previous study [14].

### Statistical analyses

Continuous variables are described using medians and interquartile ranges (IQR), and categorical variables are described using absolute counts and percentages (%). The Markov chain Monte Carlo method for multiple imputation was used to complement the missing data concerning explanatory variables used in calculating the propensity score and in the logistic regression analyses described below. Confidence intervals (CI) were estimated based on five datasets generated by multiple imputation. The enrolled patients were followed up for 30 days after cardiac arrest.

First, to compare lidocaine and amiodarone in patients with OHCA due to shockable rhythms, propensity score matching (PSM) was used to adjust for multiple confounding factors. The presence of lidocaine administration was used as a response variable for calculating the propensity scores. The following explanatory variables (i.e., variables that could influence the response variable) were identified based on previous studies [18]: age, sex, transportation by road or air ambulance with a physician present, witness status (none, EMS personnel, others), bystander CPR, the initial cardiac rhythm monitored (VF, pulseless VT, PEA, asystole, other), the cause of cardiac arrest (cardiogenic, presumed cardiogenic), the time from the emergency call to EMS personnel contact, and the time from the emergency call to physician contact. After calculating the propensity score, we confirmed that there was sufficient overlap in the distribution of the propensity scores (PSs) in the two groups with regard to lidocaine administration. For performing PSM, lidocaine and amiodarone were matched in a 1:3 ratio, and the caliper width was set to 20% of the standard deviation of the PS. After PSM, we confirmed that the confounding factors relevant to both groups were balanced using standardized differences. A value of 0.1 was used as the cut-off value of standardized differences [19].

Second, logistic regression analysis was conducted to adjust for other confounding factors not used for calculating the propensity score. We evaluated 30-day survival and good neurological outcomes 30 days after cardiac arrest as the primary response variables. The following explanatory variables (i.e., variables that could potentially influence these response variables) were identified based on the findings of a previous study [18]: administration of magnesium, nifekalant, or adrenaline; undergoing shock delivery; and use of ECMO, IABP, PCI, or TTM. Effect estimates were described using odds ratio (OR) and 95% CI.

EZR (version 1.38) and R statistical software (version 3.5.2) (The R Project for Statistical Computing, Vienna, Austria), as well as SPSS statistical software (version 26, IBM, Armonk, NY, USA), were used to conduct the present analysis. A two-sided *p*-value of < 0.05 was considered the threshold for statistical significance.

## Results

**Fig. 1:**
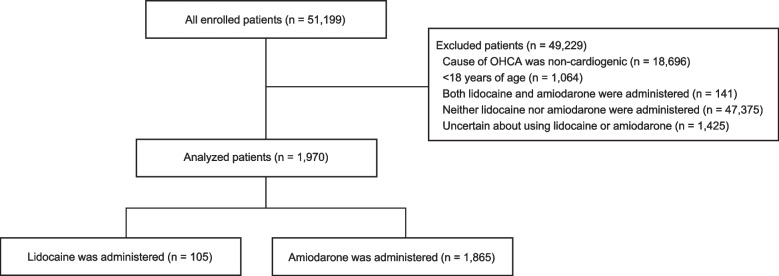
Flowchart depicting the screening and enrolment process within this study. OHCA, out-of-hospital cardiac arrest

Of 51,199 patients registered to the OHCA registry, we analyzed data of 1970 patients who were administered only lidocaine or amiodarone (Fig. 1). The reasons for exclusion were as follows: 18,696 patients for the cause of OHCA being non-cardiogenic, 1064 patients for being < 18 years of age, 141 patients for whom both lidocaine and amiodarone were administered, 47,375 patients for whom neither lidocaine nor amiodarone was administered, and 1425 patients for whom there was uncertainty about their use of lidocaine or amiodarone.

Patient characteristics are shown in Table 1. Overall, the median age (IQR) was 66 (55–75) years, and 1606 patients (81.5%) were male; 811 patients (41.2%) had their cardiac arrest witnessed by EMS personnel, whereas 625 patients (31.7%) had their cardiac arrest witnessed by others. Moreover, 946 patients (48.0%) underwent bystander-initiated CPR; 1209 patients (61.4%) had VF as their initial monitored cardiac rhythm, whereas 12 patients (0.6%) presented with pulseless VT, and 305 patients (15.5%) presented with PEA. Also, 18,500 patients (95.0%) experienced shock delivery; 105 patients (5.3%) were administered lidocaine, and 1865 patients (94.7%) were administered amiodarone. The median time from the emergency call to EMS contact was 8 (6–10) min, the median time from the emergency call to physician contact was 31 (25–38) min, and the median time from the emergency call to ROSC was 43 (27–62) min; 475 patients (24.1%) had survived as of 30 days after cardiac arrest, and 262 patients (13.3%) had good 30-day neurological outcomes. Missing data are described in Table 2.

**Table 1:** Baseline characteristics and comparison of lidocaine and amiodarone

	Before matching				After matching (before multiple imputations)			
Variables	Overall (*n* = 1970)	Lidocaine (*n* = 105)	Amiodarone (*n* = 1865)	SD	Overall (*n* = 252)	Lidocaine (*n* = 63)	Amiodarone (*n* = 189)	SD
Age (years)	66 (55–75)	69 (61–79)	66 (54–75)	0.28	69 (60–79)	68 (59.5–79.5)	69 (60–78)	0.01
Male, n (%)	1606 (81.5)	81 (77.1)	1525 (81.8)	0.12	192 (76.2)	49 (77.8)	143 (75.7)	0.05
Transportation by vehicular or air ambulance with a physician, n (%)	416 (78.9)	12 (11.4)	404 (21.7)	0.28	27 (10.7)	7 (11.1)	20 (10.6)	0.02
Witness status, n (%)				0.20				0.05
EMS personnel	811 (41.2)	51 (48.6)	760 (40.8)		110 (43.7)	27 (42.9)	83 (43.9)	
Others	625 (31.7)	25 (23.8)	600 (32.2)		74 (29.4)	18 (28.6)	56 (29.6)	
Bystander CPR, n (%)	946 (48.0)	46 (43.8)	900 (48.3)	0.09	114 (45.2)	29 (46.0)	85 (45.0)	0.02
Initial cardiac rhythm monitored, n (%)				0.35				0.21
VF	1209 (61.4)	49 (46.7)	1160 (62.2)		127 (50.4)	30 (47.6)	97 (51.3)	
Pulseless VT	12 (0.6)	1 (0.01)	11 (0.6)		0 (0)	0 (0)	0 (0)	
PEA	305 (15.5)	24 (22.9)	281 (15.1)		61 (24.2)	14 (22.2)	47 (24.9)	
Asystole	329 (16.7)	26 (24.8)	303 (16.2)		58 (23.0)	16 (25.4)	42 (22.2)	
Others	115 (5.8)	5 (4.8)	110 (5.9)		6 (2.4)	3 (4.8)	3 (1.6)	
Cause of cardiac arrest, n (%)				0.03				0.10
Cardiogenic	248 (19.2)	12 (11.4)	236 (19.3)		37 (14.7)	11 (17.5)	26 (13.8)	
Presumed cardiogenic	1043 (80.8)	54 (51.4)	989 (80.7)		215 (85.3)	52 (82.5)	163 (86.2)	
Presence of shock delivery, n (%)	1850 (95.0)	83 (79.1)	1767 (95.6)	0.42	225 (91.1)	49 (81.7)	176 (94.1)	0.39
Administration of adrenaline, n (%)	1748 (88.9)	88 (83.8)	1660 (89.2)	0.13	225 (89.3)	51 (81.0)	174 (92.1)	0.33
Administration of antiarrhythmic drugs, n (%)								
Lidocaine	105 (5.3)	105 (100)	0 (0)	–	63 (25.0)	63 (100)	0 (0)	–
Amiodarone	1865 (94.7)	0 (0)	1865 (100)		189 (75.0)	0 (0)	189 (100)	–
Nifekalant	69 (3.5)	5 (4.8)	64 (3.4)	0.07	5 (2.0)	2 (3.2)	3 (1.6)	0.10
Magnesium	238 (12.1)	9 (8.6)	229 (12.3)	0.12	27 (10.7)	2 (3.2)	25 (13.2)	0.37
Time from call to physician contact, mins	31 (25–38)	29 (24–38)	31 (25–38)	0.06	31 (26–38)	29 (24–39.5)	32 (27–38)	0.03
Time from call to EMS contact, mins	8 (6–10)	8 (6–10)	8 (7–10)	0.14	8 (7–10)	8 (7–10)	8 (7–10)	0.03
Time from call to ROSC, mins	43 (27–62)	36 (27.3–52.8)	43 (27–63)	0.15	39 (26–58.5)	34 (23–49.5)	41.5 (26.8–60)	0.38
Types of treatment, n (%)								
ECMO	884 (44.9)	26 (24.8)	858 (46.0)	0.46	98 (38.9)	17 (27.0)	81 (42.9)	0.34
IABP	695 (35.3)	21 (20.0)	674 (36.1)	0.37	74 (29.4)	12 (19.0)	62 (32.8)	0.32
PCI	558 (28.3)	23 (21.9)	535 (28.7)	0.16	65 (25.8)	16 (25.4)	49 (25.9)	0.01
TTM	642 (32.6)	27 (25.7)	615 (33.0)	0.16	76 (30.2)	19 (30.2)	57 (30.2)	< 0.001
30-day CPC, n (%)								
1, good cerebral recovery	194 (9.8)	11 (10.5)	183 (9.8)		17 (6.7)	7 (11.1)	10 (5.3)	
2, moderate cerebral disability	68 (3.5)	4 (3.8)	64 (3.4)		7 (2.8)	3 (4.8)	4 (2.1)	
3, severe cerebral disability	83 (4.2)	3 (2.9)	80 (4.3)		9 (3.6)	2 (3.2)	7 (3.7)	
4, coma or vegetative state	130 (6.6)	6 (5.7)	124 (6.6)		17 (6.7)	5 (7.9)	12 (6.3)	
5, death or brain death	1495 (75.9)	81 (77.1)	1414 (75.8)		202 (80.2)	46 (73.0)	156 (82.5)	
30-day survival, n (%)	475 (24.1)	24 (22.9)	451 (24.2)		54 (21.1)	17 (26.6)	37 (19.3)	
30-day good neurological outcome, n (%)	262 (13.3)	15 (14.3)	247 (13.2)		24 (9.5)	10 (15.9)	14 (7.4)	

Data are presented as medians (interquartile ranges) for continuous variables and numbers (proportions) for categorical values. We conducted a complete case analysis

*Abbreviations*: *CPC* Cerebral Performance Category; *CPR* Cardiopulmonary Resuscitation; *ECMO* Extracorporeal Membrane Oxygenation; *EMS* Emergency Medical Services; *IABP* Intra-Aortic Balloon Pumping; *PCI* Percutaneous Coronary Intervention; *PEA* Pulseless Electrical Activity; *ROSC* Return of Spontaneous Circulation; *SD* Standardized Difference; *TTM* Targeted Temperature Management; *VF* Ventricular Fibrillation; *VT* Ventricular Tachycardia

**Table 2:** Number of missing data of the characteristics of analyzed patients

Variables, n (%)	Overall (*n* = 1970)	Lidocaine (*n* = 105)	Amiodarone (*n* = 1865)
Cause of cardiac arrest	679 (34.5)	39 (37.1)	640 (34.3)
Presence of shock delivery	22 (1.0)	5 (4.8)	17 (0.9)
Administration of adrenaline	3 (0.2)	0 (0)	3 (0.2)
Administration of magnesium	11 (0.6)	2 (1.9)	9 (0.5)
Time from call to physician contact	9 (0.5)	0 (0)	9 (0.5)
Time from call to ROSC	709 (36.0)	35 (33.3)	674 (36.1)

Data are presented as numbers (proportions)

*Abbreviation*: *ROSC* Return of Spontaneous Circulation

After performing PSM (i.e., before multiple imputation), the standardized differences of the variables used in the PSM were all < 0.1 (Table 1). After performing multiple imputation and logistic regression analysis, the ORs and 95% CIs concerning the use of lidocaine in evaluating 30-day survival and good neurological outcomes were 1.44 (0.58–3.61) and 1.77 (0.59–5.29), respectively (Table 3).

**Table 3:** Odds ratio for lidocaine or amiodarone use in patients with OHCA

		Propensity score matching		
		Odds ratio	95% CI (lower)	95% CI (upper)
30-day survival	Amiodarone	Reference		
	Lidocaine	1.44	0.58	3.61
30-day good neurological outcome^a^	Amiodarone	Reference		
	Lidocaine	1.77	0.59	5.29

*Abbreviations*: *CI* Confidence Interval; *CPC* Cerebral Performance Category; *OHCA* Out-of-Hospital Cardiac Arrest

^a^A good neurological outcome was defined as a CPC score of 1 or 2

## Discussion

In our study, only 5.3% of patients with OHCA due to shockable rhythms were administered lidocaine, whereas 94.7% were administered amiodarone. There were no significant differences in both 30-day survival or good neurological outcomes between the two groups.

The group of patients administered lidocaine was smaller than amiodarone since a limited amount of time had passed from the results of the aforementioned RCT, published in 2016 [14]. The data registration period of the JAAM-OHCA registry was between June 1, 2014, and December 31, 2019, and it was only approximately three years from the publication of RCT to the end of the registration period [14]. In general, RCTs need to be carefully validated for external validity, and this recognition is spreading worldwide [20]. Therefore, time might be needed to validate the external validity of the RCT before applying the results to real-world clinical practice, and approximately three years might be too short to validate and use the RCT results.

The findings of our study are similar to those of the aforementioned RCT, comparing lidocaine and amiodarone use in patients with OHCA [14]. There is a possible reason for the lack of significant differences between the lidocaine and amiodarone groups in this study. The characteristics of patients in our study were not appropriate for detecting differences between lidocaine and amiodarone use. More specifically, after matching, almost 50% of patients’ initial cardiac rhythms were identified as asystole or PEA, and almost 85% of patients’ causes of cardiac arrest were presumed to be of cardiac origin. Since the cause of arrhythmia was not cardiogenic, we suggest that the evaluated antiarrhythmic drugs might not be sufficiently effective for this population. If a more accurate extraction method for patients with OHCA due to cardiogenic causes is devised in the future, and if future studies are conducted using such an extraction method, different results may be obtained.

Our study is novel in that, although patients’ respective characteristics and outcomes were found to be similar to those reported within a previous RCT, our study verified the external validity of these prior findings through confirmation in a real-world setting [14]. According to our findings, as well as those of the prior RCT, there is no evidence that either amiodarone or lidocaine is superior in the treatment of patients with OHCA due to shockable rhythms; thus, either or both drugs could be administered in treating this condition.

However, we acknowledge several limitations of our study. First, the characteristics of the eligible patients might be inappropriate for evaluating the effects of antiarrhythmic drugs. In the JAAM-OHCA registry, the cause of cardiac arrest (cardiogenic or noncardiogenic) is often recorded soon after arrival at the hospital and before a definitive diagnosis is made. In addition, there were no data on changes in cardiac rhythm during transport and after arrival at the hospital. In our study, approximately 30% of initial cardiac rhythms were PEA or asystole. Unfortunately, detailed rhythm changes information for these patients were not available. Therefore, our study could not accurately identify a group of patients for whom antiarrhythmic drugs are effective, and we might not have detected differences. However, in actual clinical practice, antiarrhythmic drugs are used even when the cardiac arrest cause is unknown or when cardiac rhythm changes from non-shockable rhythm to VF or pulseless VT, and the analysis of our study was performed under actual clinical practice.

Second, the 95% CIs obtained in our study were wide and imprecise, and the logistic regression results might be unreliable. In our study, only 5.3% of patients were administered lidocaine, and few patients could be included in multivariate-adjusted analyses. Therefore, the 95% CI was even wider within this subgroup, and the validity of the analysis results decreased further as a result.

Finally, since there were no data on the dosage, frequency, and timing of the administration of either amiodarone or lidocaine in this registry, higher doses of antiarrhythmic drugs are generally expected to have greater antiarrhythmic effects and might therefore be expected to affect clinical outcomes. Furthermore, the time from onset of arrhythmia or ROSC to drug administration might also be a confounding factor.

## Conclusions

Our study showed no significant differences between lidocaine and amiodarone use in patients with OHCA due to shockable rhythms for 30-day survival or 30-day neurological outcomes within a real-world setting. Although this result needs to be evaluated more comprehensively within future highly powered investigations, there is no evidence that either amiodarone or lidocaine is superior in the treatment of patients with OHCA due to shockable rhythms, and thus, either or both drugs could be administered for the treatment.

## Supplementary Information

**Additional file 1: e-Table 1.** STROBE Statement—checklist of items that should be included in reports of observational studies.

**Additional file 2: e-Table 2.** List of ethics committee of participating institutions of Japanese Association for Acute Medicine- out-of-hospital cardiac arrest registry.

## Data Availability

The use of JAAM-OHCA registry data is limited to members of the Japanese Association of Acute Medicine, and permission must be obtained from the society. We do not wish to share the data of our study, since it is not authorized by the Commission of JAAM-OHCA registy.

## Abbreviations

AHA: American Heart Association
CI: Confidence Interval
CPC: Cerebral Performance Category
CPR: Cardiopulmonary Resuscitation
ECMO: Extracorporeal Membrane Oxygenation
EMS: Emergency Medical Services
ERC: European Resuscitation Council
IABP: Intra-Aortic Balloon Pumping
IQR: Interquartile Range
JAAM: Japanese Association for Acute Medicine
OHCA: Out-of-Hospital Cardiac Arrest
OR: Odds Ratio
PCI: Percutaneous Coronary Intervention
PEA: Pulseless Electrical Activity
PSM: Propensity Score Matching
PS: Propensity Score
RCT: Randomized Control Trial
ROSC: Return of Spontaneous Circulation
SD: Standardized Difference
STROBE: STrengthening the Reporting of OBservational studies in Epidemiology
TTM: Targeted Temperature Management
VF: Ventricular Fibrillation
VT: Ventricular Tachycardia
