# Supplementary material for: Comparison of the effects of lidocaine and amiodarone for out-of-hospital cardiac arrest patients with shockable rhythms: a retrospective observational study from a multicenter registry
Source: BMC Cardiovasc Disord. 2022 Nov 5;22:466. doi: 10.1186/s12872-022-02920-2 (PMC9636706; doi:10.1186/s12872-022-02920-2)
Supplement: Supplementary file 2 — Additional file 2: e-Table 2. List of ethics committee of participating institutions of Japanese Association for Acute Medicine- out-of-hospital cardiac arrest registry. [file 12872_2022_2920_MOESM2_ESM.docx]

**e-Table 2. List of ethics committee of participating institutions of Japanese Association for Acute Medicine- out-of-hospital cardiac arrest registry**

| The name of institutions |
| --- |

| Ethics Committee of Akita University Hospital |
| --- |
| Ethics Committee of Asahikawa Medical University Hospital |

| Ethics Committee of Center Hospital of the National Center for Global Health and Medicine |
| --- |

| Ethics Committee of Chiba University Hospital |
| --- |
| Ethics Committee of Ehime University Hospital |
| Ethics Committee of Fujisawa City Hospital |
| Ethics Committee of Fujieda Municipal General Hospital |
| Ethics Committee of Fukuyama City Hospital |
| Ethics Committee of Gifu Prefectural General Medical Center |
| Ethics Committee of Gifu University Hospital |
| Ethics Committee of Gunma University Hospital |
| Ethics Committee of Hachinohe City Hospital |
| Ethics Committee of Hakodate Municipal Hospital |
| Ethics Committee of Hamamatsu University Hospital |
| Ethics Committee of Hokkaido Medical Center National Hospital |

| Ethics Committee of Hokkaido University Hospital |
| --- |
| Ethics Committee of Hospital of the University of Occupational and Environmental Health |

| Ethics Committee of Hyogo Emergency Medical Center |
| --- |
| Ethics Committee of Hyogo Prefectural Kobe Children's Hospital |
| Ethics Committee of Ibaraki Prefectural Central Hospital, Ibaraki Cancer Center |
| Ethics Committee of Iwate Medical University Hospital |

| Ethics Committee of Iwate Prefectural Central Hospital |
| --- |

| Ethics Committee of Japanese Red Cross Maebashi Hospital |
| --- |

| Ethics Committee of Japanese Red Cross Saitama Hospital |
| --- |
| Ethics Committee of Japanese Red Cross Society Kyoto Daini Hospital |
| Ethics Committee of Jichi Medical University Saitama Medical Center |

| Ethics Committee of Kagawa University Hospital |
| --- |

| Ethics Committee of Kansai Medical University Hospital |
| --- |

| Ethics Committee of Kansai Medical University Medical Center |
| --- |
| Ethics Committee of Kimitsu Chuo Hospital |

| Ethics Committee of Kindai University Hospital |
| --- |

| Ethics Committee of Kin-ikyo Chuo Hospital |
| --- |
| Ethics Committee of Kishiwada Tokushukai Hospital |
| Ethics Committee of Kobe City Medical Center General Hospital |
| Ethics Committee of Kumamoto University Hospital |
| Ethics Committee of Kyoto City Hospital |

| Ethics Committee of Kyoto University Hospital |
| --- |

| Ethics Committee of Kyushu University Hospital |
| --- |
| Ethics Committee of Miyazaki Prefectural Nobeoka Hospital |
| Ethics Committee of Mizushima Central Hospital |
| Ethics Committee of Nagoya university Hospital |
| Ethics Committee of National Center for Child Health and Development |
| Ethics Committee of National Cerebral and Cardiovascular Center |
| Ethics Committee of National Hospital Organization Kumamoto Medical Center |
| Ethics Committee of National Hospital Organization Mito Medical Center |
| Ethics Committee of National Hospital Organization Nagasaki Medical Center |

| Ethics Committee of National Hospital Organization Osaka National Hospital |
| --- |

| Ethics Committee of National Hospital Organization Takasaki General Medical Center |
| --- |
| Ethics Committee of Nihon University Hospital |

| Ethics Committee of Nihon University Itabashi Hospital |
| --- |
| Ethics Committee of Niigata Prefectural Hospital |
| Ethics Committee of Niigata University Medical & Dental Hospital |
| Ethics Committee of Nagasaki University Hospital |
| Ethics Committee of Nihonkai General Hospital |
| Ethics Committee of Nippon Medical School Musashi Kosugi Hospital |

| Ethics Committee of Nippon Medical School Tama Nagayama Hospital |
| --- |
| Ethics Committee of Okayama University Hospital |
| Ethics Committee of Okinawa Prefectural Chubu Hospital |

| Ethics Committee of Osaka City General Hospital |
| --- |
| Ethics Committee of Osaka General Medical Center |
| Ethics Committee of Osaka Metropolitan University Hospital |
| Ethics Committee of Osaka Police Hospital |
| Ethics Committee of Osaka Prefectural Nakakawachi Emergency and Critical Care Center |

| Ethics Committee of Osaka Red Cross Hospital |
| --- |

| Ethics Committee of Osaka University Hospital |
| --- |
| Ethics Committee of Rinku General Medical Center |

| Ethics Committee of Saga Medical School Hospital |
| --- |

| Ethics Committee of Saiseikai Senri Hospital |
| --- |

| Ethics Committee of Saitama Medical University International Medical Center |
| --- |
| Ethics Committee of Sapporo Higashi Tokushukai Hospital |

| Ethics Committee of Sapporo Medical University |
| --- |

| Ethics Committee of Seirei Hamamatsu General Hospital |
| --- |
| Ethics Committee of Shinshu University Hospital |
| Ethics Committee of SHIN-YURIGAOKA General Hospital |
| Ethics Committee of Showa University Hospital |

| Ethics Committee of St. Luke's International Hospital |
| --- |

| Ethics Committee of Takarazuka Hospital |
| --- |

| Ethics Committee of Tane General Hospital |
| --- |

| Ethics Committee of Teine Keijinkai Hospital |
| --- |
| Ethics Committee of The University of Tokyo Hospital |
| Ethics Committee of Tohoku Medical and Pharmaceutical University Hospital |

| Ethics Committee of Tohoku University Hospital |
| --- |
| Ethics Committee of Tokyobay UrayasuIchikawa Medical Center |
| Ethics Committee of Tokyo Medical And Dental University Hospital |

| Ethics Committee of Tokyo Metropolitan Children’s Medical Center |
| --- |
| Ethics Committee of University Hospital Kyoto Prefectural University of Medicine |
| Ethics Committee of Yamagata Prefectural Central Hospital |

| Ethics Committee of Yamaguchi University Hospital |
| --- |

| Ethics Committee of Yamanashi Prefectural Central Hospital |
| --- |
| Ethics Committee of Yokohama City Minato Red Cross Hospital |

| Ethics Committee of Yokohama City University Medical Center |
| --- |
